# 947 Burns Associated with Home Oxygen Therapy - A Regional and National Study

**DOI:** 10.1093/jbcr/iraf019.478

**Published:** 2025-04-01

**Authors:** John Warner-Levy, Zeeshan Sheikh, Karl Walsh

**Affiliations:** The University of Manchester; Manchester University NHS Foundation Trust; Manchester University NHS Foundation Trust

## Abstract

**Introduction:**

Amid increasing calls for regulation, a single-country report found that between August 2019 and July 2021, 256 incidents and 152 deaths were linked to home oxygen therapy-related fires. In a 2022 statement, the ABA expressed support for legislative efforts to reduce burn injuries, advocating for the mandated use of bidirectional thermal fuses (firebreaks) in oxygen tubing, aligning with ISO 80601-2-69:2020. This was supported by a 2024 study, which concluded that firebreaks are cost-effective in preventing burn-related morbidity, mortality, and property damage. Subsequently, we conducted a service evaluation to assess the efficacy of these measures at our regional Burns Centre, where firebreaks have been mandated since 2005.

**Methods:**

A retrospective review of patients treated at a large hospital system from January 2005 to August 2024 was conducted to identify burn incidents associated with the use of home oxygen therapy. Subsequently, national data for the same period was requested from iBID.

**Results:**

Our local search identified 41 cases of burns related to home oxygen therapy, with males comprising 56.1% of cases and a mean patient age of 62.9 years (SD 10.0). The average TBSA was 1.9% (SD 4.0), with burn depth predominantly superficial (65.7%), followed by mid-depth (31.4%) and deep burns (2.9%). Most incidents (89.7%) occurred in the patient’s home, of which 53.6% were in the living room. Most injuries (59.1%) healed within a week, with an average hospital stay of 9.6 days (SD 12.4), and one patient requiring skin grafting. Two patients (4.9%) died as a result of their injuries. Nationally, an extra 330 home oxygen therapy-associated burns were recorded in the same period.

**Conclusions:**

Our findings indicate that home oxygen therapy-related burns continue to occur, despite existing regulations. A possible factor is the removal or improper assembly of firebreaks by patients. We believe it is crucial to emphasise patient education on correctly installing and maintaining firebreaks. Additionally, starting in the late 2010s, firebreaks transitioned from one-way to two-way valves. This shift, combined with past instances of improper installation, may still present ongoing issues.

**Applicability of Research to Practice:**

New regulations, perhaps surrounding non-removable firebreaks, could reduce the incidence of this rare but potentially devastating form of burn.

**Funding for the Study:**

This research received no specific grant from funding agencies in the public, commercial, or not-for-profit sectors.

